# TGF-β1 Upregulates the Expression of Triggering Receptor Expressed on Myeloid Cells 1 in Murine Lungs

**DOI:** 10.1038/srep18946

**Published:** 2016-01-07

**Authors:** Li Peng, Yong Zhou, Liang Dong, Rui-Qi Chen, Guo-Ying Sun, Tian Liu, Wen-Zhuo Ran, Xiang Fang, Jian-Xin Jiang, Cha-Xiang Guan

**Affiliations:** 1 Department of Physiology, Xiangya School of Medicine, Central South University, Changsha, China; 2 Department of Neurology, University of Texas Medical Branch, Galveston, TX 77555, USA; 3 State Key Laboratory of Trauma, Burns, and Combined Injury, Research Institute of Surgery, Daping Hospital, Third Military Medical University, Chongqing, China

## Abstract

Triggering receptor expressed on myeloid cells 1 (TREM-1) increases the expression of TGF-β family genes, which are known as profibrogenic cytokines in the pathogenesis of pulmonary fibrosis. In this study, we determined whether TGF-β1 regulated the expression of TREM-1 in a mouse model of pulmonary fibrosis. The expression of TGF-β1 and TREM-1 was increased on day 7, 14, and 21 after single intratracheal injection of bleomycin (BLM). And there was positive correlation between the expression of TGF-β1 and TREM-1. TGF-β1 increased expression of TREM-1 mRNA and protein in a time- and dose-dependent manner in mouse macrophages. The expression of the activator protein 1 (AP-1) was increased in lung tissues from mouse after BLM injection and in mouse macrophages after TGF-β1 treatment, respectively. TGF-β1 significantly increased the relative activity of luciferase in the cells transfected with plasmid contenting wild type-promoter of TREM-1. But TGF-β1 had no effect on the activity of luciferase in the cells transfected with a mutant-TREM1 plasmid carrying mutations in the AP-1 promoter binding site. In conclusion, we found the expression of TREM-1 was increased in lung tissues from mice with pulmonary fibrosis. TGF-β1 increased the expression of TREM-1 in mouse macrophages partly via the transcription factor AP-1.

The term “genomic storm” describes a new paradigm in human immune and inflammatory responses at the time of serious injury^1^. Triggering receptor expressed on myeloid cells 1 (TREM-1) expression is increased significantly during “genomic storms”^2^. As a cell-membrane surface receptor belonging to the IgG super family, TREM-1 is selectively expressed in mononuclear macrophages and neutrophils^3^,^4^. Synergistic activation of TREM-1, toll-like receptor, and nod-like receptor results in the activation of pro-inflammatory factors such as TNF-α and IL-1β, as well as the inhibition of the anti-inflammatory factor IL-10^5^, which prolongs macrophage survival^6^ and ultimately leads to excessive inflammatory responses. TREM-1 is reported to amplify inflammatory responses and aggravate acute lung injury and acute respiratory distress syndrome^4^,^7^. However, there is little research on the role of TREM-1 in fibrotic disease. A recent study found that the expression of TREM-1 was significantly increased in renal fibrosis^8^, while our previous observations also shown that TREM-1 was expressed in lung tissues^4^. It has been shown that TREM-1 activation increases the expression of transforming growth factor (TGF)-β family genes by 96.7-fold^9^. These previous findings prompted us to speculate that there is a putative link between TREM-1 and pulmonary fibrosis.

Pulmonary fibrosis is a chronic, progressive and devastating interstitial lung disease^10^,^11^,^12^. Clinically, pulmonary fibrosis manifests as a continuous decline in lung function, which eventually leads to respiratory failure^13^. The morbidity and mortality of pulmonary fibrosis, which is similar to lung cancer, are increasing^14^. The median survival period of pulmonary fibrosis is 2–5 years after diagnosis^15^, and patients often have poor prognosis and impairment of health-related quality of life. Currently, there is no effective drug treatment for late stage pulmonary fibrosis, and the exponential increase in the number of clinical trials for the treatment of pulmonary fibrosis has shown few promising results^16^. Although the origin and pathogenesis of pulmonary fibrosis is complex, TGF-β1 has been implicated as a key player in the pathogenesis of pulmonary fibrosis, as it may induce proliferation and differentiation of fibroblasts, epithelial-mesenchymal transition (EMT), and transformation of lung fibroblasts to myofibroblasts, which eventually lead to serious pulmonary fibrosis *in vivo and in vitro*^17^,^18^,^19^.

TGF-β1 is a central node in the pulmonary fibrosis pathogenesis pathway, while TREM-1 activation dramatically increases the expression of TGF-β family genes. Conversely, it remains unknown whether the increase of TGF-β1 could affect the expression of TREM-1. This study was designed to determine the effect of TGF-β1 on the expression of TREM-1 in lungs and to explore the transcriptional mechanism at both the organismal and cellular levels, which will provide novel insights into the regulatory mechanisms in the pathogenesis of pulmonary fibrosis, and therefore the potential therapeutic targets for the treatment of pulmonary fibrosis.

## Materials and Methods

### Establishment of a Mouse Pulmonary Fibrosis Model

All animal care and experimental protocols were in compliance with the Animal Management Rules of the Ministry of Health of the People’s Republic of China. The animal experimental protocols were approved by the Ethics Committee of Xiangya Hospital of Central South University. All mouse experiments were performed under anesthesia, and we made every effort to minimize pain during the experiments.

Male Swiss mice weighing 20 ± 2 g were purchased from the Laboratory Animal Department of Central South University, fed in a clean animal room maintained at 23 to 25 °C, 50% to 60% relative humidity, and 12 h circadian rhythm. Mice were randomly divided into two groups (n = 24/group): bleomycin (BLM)-treated and control. The BLM-treated group was intratracheally administered with BLM (5 mg/kg, Nippon Kayaku, Japan) to establish a model of pulmonary fibrosis, while the control group was administered with equal amount of normal saline^19^. The 8 mice of the each group were randomly selected and sacrificed respectively on day 7, 14 and 21 after administration of BLM or saline for further experiments.

### Histological Examination of Lung Tissues

The lower right lobe of the lungs was fixed in 4% paraformaldehyde solution and embedded in paraffin. Pathological changes in lung tissues and collagen deposition were observed by staining lung sections with hematoxylin-eosin (HE) staining or Masson triple staining.

### RNA Extraction and Real-Time PCR

RNA was extracted from lung tissues or mouse macrophages using the TRIzol method (Invitrogen, USA). The cDNA was synthesized using RevertAid First Strand cDNA short Kit (Thermo Scientific, USA) according to the manufacturer’s protocol. Transcriptional levels of procollagen types I and III, TGF-β1, TREM-1, and AP-1 were determined using UltraSYBR Mixture (cwbiotech, China) and a Real-time PCR Detection System (Bio-Rad, USA). The mRNA expression of our genes of interest was calculated by 2^−ΔΔCt^ method normalized to housekeeper GAPDH.

### Western Blotting

The lung tissues were homogenized, separated on 10% SDS-PAGE gel (Bio-Rad, USA), then transferred onto a PVDF membrane using the semi-dry method^20^,^21^,^22^. After blocking, membranes were incubated with mouse anti-TREM-1 antibodies (R&D Systems, USA) overnight. Horseradish peroxidase-conjugated secondary antibodies (Santa Cruz, USA) were applied and enhanced chemiluminescence to detect protein content. Images were collected using a FluorChemQ system (Alpha Innotech Corporation, USA).

### Cell Cultures

Mouse macrophage cell line RAW264.7 was purchased from Xiangya Central Experiment Laboratory of Central South University. Cells were cultured in DMEM (Sigma Chemical company, USA) supplemented with 10% Fetal Bovine Serum (HyClone, USA) and incubated at 37 °C in a 5% CO_2_ incubator.

### Primary alveolar macrophages isolation

Bronchoalveolar lavage fluid (BALF) were flushed three times with 1 mL ice-cold PBS in Swiss mice after anesthetization and arteria femoralis bloodletting^23^. Primary alveolar macrophages were isolated from BALF by centrifugation method and adherence-changing culture method^24^.

### Flow Cytometry

Mouse macrophages (a cell line and primary alveolar macrophages) were plated into 6-well cell culture plates and stimulated with 10 ng/mL TGF-β1 for various periods of time (0, 1, 2, 5, 10, 20, and 40 h). Different concentrations (0, 1, 2, 5, 10, and 20 ng/mL) of TGF-β1 were tested for their stimulatory effect on mouse macrophages at the 20 h time point.

Flow Cytometry was used for testing protein level of TREM-1 on the surface of cells^25^,^26^,^27^. After incubating cells for 30 min using mouse anti-TREM-1 antibody (Source: Monoclonal Rat IgG) (R&D Systems, USA) and chicken anti-rat IgG-FITC antibody (Santa Cruz, USA), the percentage of cells positive for TREM-1 was measured using a Moflo XDP type flow cytometry instrument (Beckman Coulter, USA), revealing the amount of surface-expressed TREM-1 protein. The data were analyzed with Summit 5.0 or FlowJo 7.6.5.

### Site-Directed Mutagenesis and Plasmid Construction

The TREM-1 promoter sequence in mouse found by analyzing the sequence of chromosome 17 and the EST database. The TREM-1 promoter sequence was amplified by PCR from mouse genomic DNA using Pfu polymerase (Sangon, China) and the following oligonucleotide PCR amplification primers: sense primer, 5′-caaGGTACCTGTATGTGGGCAAATGTAGTGTGTGTGGCGGG-3′ and anti-sense primer, 5′-cacacgtctcAAGCTTCCTTCAAGCTCAGCTCCAACGACTGCCTCTG-3′. PCR products were digested with KpnI and HindIII and subcloned into the firefly luciferase expression plasmid pGL3-basic (Promega, USA) to produce the wild type (WT)-TREM1 plasmid.

The TFSEARCH database was used to find transcriptional factor binding sites located within the TREM-1 5′-upstream promoter region. The promoter sequence was found to contain two AP-1 binding sites, which were mutated to adenines via site directed mutagenesis. The WT-TREM1 plasmid was used as the template with Pfu polymerase (Sangon, China) and site-directed mutagenesis oligonucleotide primers (Table 1) for PCR amplification of the entire plasmid. PCR products were purified using E.Z.N.A.TM Gel Extraction Kit (Omega, USA), then ligated with T4 DNA ligase (Thermo Scientific, USA).

Construction of the plasmid containing wild type (WT) or mutation of the activator protein 1 (AP-1) promoter sequence using site-directed mutagenesis were based on the Hosoda study (Table 1)^28^. WT-TREM-1 and mutant-TREM-1 plasmid sequences were confirmed using BigDye^®^ Terminator v3.1 sequencing kit and 3730xl DNA Analyzer (Applied Biosystems, USA).

### Transfection and Dual-Luciferase Reporter Assay

Mouse macrophages (1 × 10^5^) per well were plated into a 24-well cell culture plate, transfected with either the firefly luciferase expression plasmid pGL3-basic (500 ng), WT-TREM-1 (500 ng), or mutant-TREM-1 (500 ng). The renilla luciferase expression plasmid pRL-TK (1 ng) (Promega, USA) was transfected into all cells as an internal control. All transfections were performed using Lipofetamine^®^ 2000 (Invitrogen, USA) and cells were incubated for 5 h following transfection. Thereafter, mouse macrophages were stimulated with TGF-β1 (10 ng/mL) for 20 h, washed twice with PBS, and lysed in 100 uL Passive Lysis Buffer (Promega, USA). Following this, firefly and renilla luciferase activities were detected using a Dual-luciferase^®^ Reporter Assay System (Promega, USA) and Varioskan Flash (Thermo SCIENTIFIC, USA). Relative luciferase activities were standardized to renilla luciferase activity and expressed as a ratio of firefly luciferase activity to renilla luciferase activity.

### Statistical Analysis

The data were analyzed using SPSS 17.0 software and expressed as mean ± standard deviation (SD). The difference between two groups was tested using the t-test, while difference between multiple groups was tested using one-way analysis of variance. The SNK test served as the post hoc test for multiple comparisons. Correlations were analyzed using Pearson Correlation analysis. A *p*-value < 0.05 was considered to be statistically significant.

## Results

### Establishment of Pulmonary Fibrosis Model in Mice

To verify the establishment of our mouse pulmonary fibrosis model, morphological changes in the mouse lung tissues was observed. HE staining results showed that lung tissues in mice from the control group had intact structure, clear alveolar outlines, thin alveolar septum, and no sign of inflammation (Fig. S1a–c). In the BLM-treated group, there was extensive infiltration of inflammatory cells on day 7 (Fig. S1d). The destruction of alveolar structure, formation of fibrotic lesions, and persistent inflammation were present on day 14 (Fig. S1e). Further serious damage to the alveolar structure and extensive fibrosis were observed on day 21 (Fig. S1f). The mRNA levels of procollagen types I and III in lung tissues were dramatically ascended on day 14 and 21 when compared to control group (*p* < 0.05) (Fig. S1m,n). The deposition of collagen in lung tissues was detected by Masson staining, and we found the quantity of collagen increased dramatically and extensive pulmonary fibrosis was formed on days 7, 14 and 21 in mice from the BLM-treated group in comparison with the control group (Fig. S1g–l).

### TGF-β1 Expression Positively Correlated with TREM-1 Overexpression in Pulmonary Fibrosis

**Figure 1:**
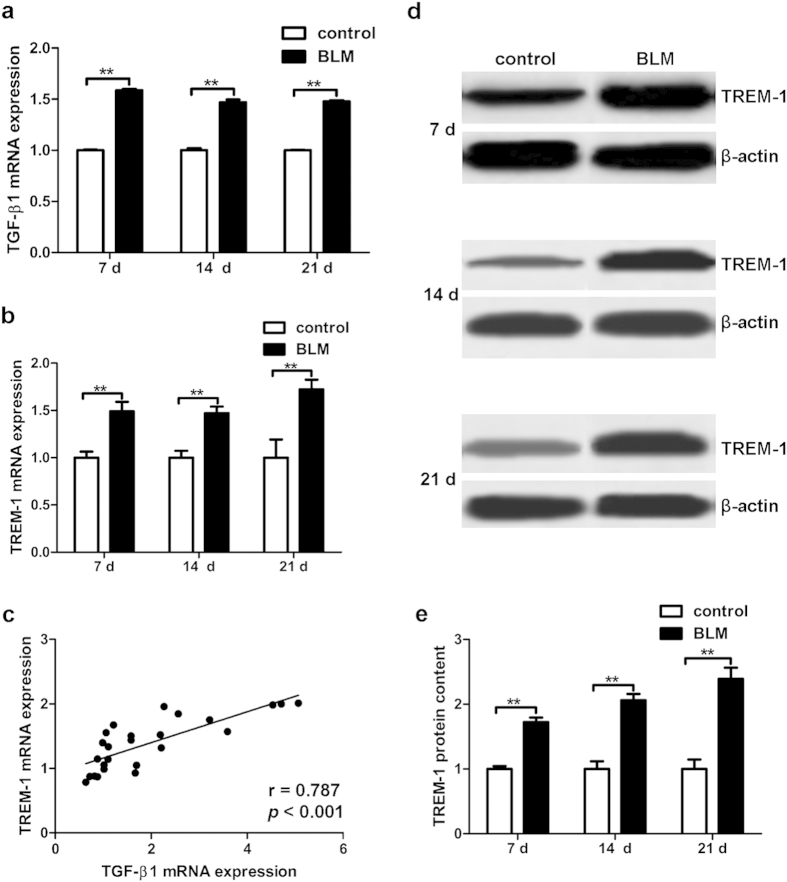
TGF-β1 expression was positive correlated with TREM-1 upregulation in mouse lungs. The mRNA levels of TGF-β1 (**a**) and TREM-1 (**b**) in mouse lungs were detected by real-time PCR. The correlation between TGF-β1 and TREM-1 (**c**) was analyzed by Pearson correlation analysis and the correlation coefficient was 0.787, p < 0.01. The controls for each time-point were normalized as 1 in (**e**). Expression of TREM-1 protein (**d**,**e**) in lung tissues was detected by Western blot. Data were expressed as the mean ± SD with 8 mice per group, **p < 0.01.

To observe the expression changes of TGF-β1 and TREM-1 in pulmonary fibrosis, TGF-β1 and TREM-1 mRNA levels were measured by real-time PCR. The results showed that both TGF-β1 and TREM-1 mRNA levels were significantly increased on day 7, 14 and 21 in the BLM-treated group (*p* < 0.05) (Fig. 1a,b). The TREM-1 protein detected by western-blot in mouse lung tissues was also remarkably increased (*p* < 0.05) (Fig. 1d,e). To further analyze the relationship between TGF-β1 and TREM-1, we applied Pearson Correlation analysis and showed that the change of TGF-β1 mRNA expression was positively correlated with change of TREM-1 mRNA expression in murine lungs (r = 0.787, *p* < 0.05) (Fig. 1c).

### TGF-β1 increased TREM-1 Expression in a Time- and Dose-Dependent Manner in Mouse Macrophages

**Figure 2:**
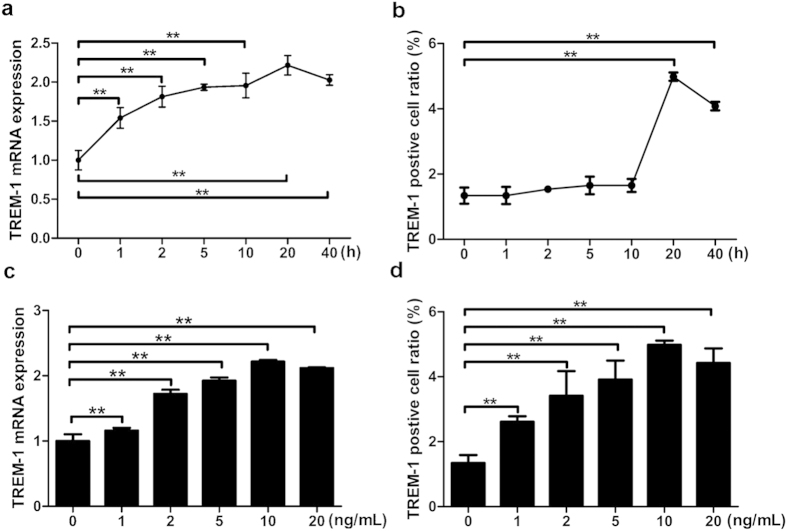
TGF-β1 increased TREM-1 expression in a time- and dose-dependent manner in mouse macrophages line. TREM-1 mRNA expression (**a**) of mouse macrophages line following stimulation with 10 ng/mL TGF-β1 for various periods of time (0, 1, 2, 5, 10, 20, and 40 h) was examined by real-time PCR. TREM-1 protein level (**b**) on the surface of mouse macrophages line after stimulation with 10 ng/mL TGF-β1 for various periods of time was determined by Flow cytometry. TREM-1 mRNA expression (**c**) of macrophages line after incubation with TGF-β1 at various concentrations (0, 1, 2, 5, 10, and 20 ng/mL) for 20 h was detected by real-time PCR. The protein content of TREM-1 (**d**) on the surface of mouse macrophages line following incubation with TGF-β1 at various concentrations for 20 h was examined by Flow cytometry. Data were expressed as mean ± SD and were representative of 5 separate experiments performed in quadruplicate, **p < 0.01.

To test whether TGF-β1 can affect the expression of TREM-1 in mouse macrophages, we measured TREM-1 mRNA level by real-time PCR after stimulation with 10 ng/mL TGF-β1 for various periods of time (0, 1, 2, 5, 10, 20, and 40 h). TREM-1 mRNA level was increased notably after treatment with TGF-β1 (10 ng/mL) for 1, 2, 5, 10, 20, and 40 h, and the most notable increase was observed at 20 h (*p* < 0.05) (Fig. 2a). The TREM-1 protein in mouse macrophages cell line was measured through Flow cytometry. These results showed that the expression of TREM-1 protein did not change significantly after 1, 2, 5, and 10 h incubation with TGF-β1 (*p* > 0.05). However, there was a significant increase in TREM-1 protein expression after 20 and 40 h, with the highest expression level of TREM-1 at 20 h after TGF-β1 stimulation in mouse macrophages cell line (*p* < 0.05) (Figs 2b, and S2a).

To assess the effect of different concentrations of TGF-β1 on TREM-1 expression, mouse macrophages cell line was incubated with TGF-β1 at various concentrations (0, 1, 2, 5, 10, and 20 ng/mL) for 20 h, and TREM-1 mRNA level was measured by real-time PCR at the end of each incubation time. Expression of the TREM-1 mRNA was increased prominently when 2, 5, 10, and 20 ng/mL TGF-β1 were used and expression of TREM-1 mRNA reached its peak level at 10 ng/mL TGF-β1 (*p* < 0.05) (Fig. 2c). TREM-1 protein in mouse macrophages cell line was detected by Flow cytometry analysis. It revealed that expression of the TREM-1 protein was increased following stimulation with all tested concentrations of TGF-β1 in a dose-dependent manner, with a peak in expression when 10 ng/mL TGF-β1 was used to stimulate mouse macrophages cell line (*p* < 0.05) (Figs 2d, and S2b).

**Figure 3:**
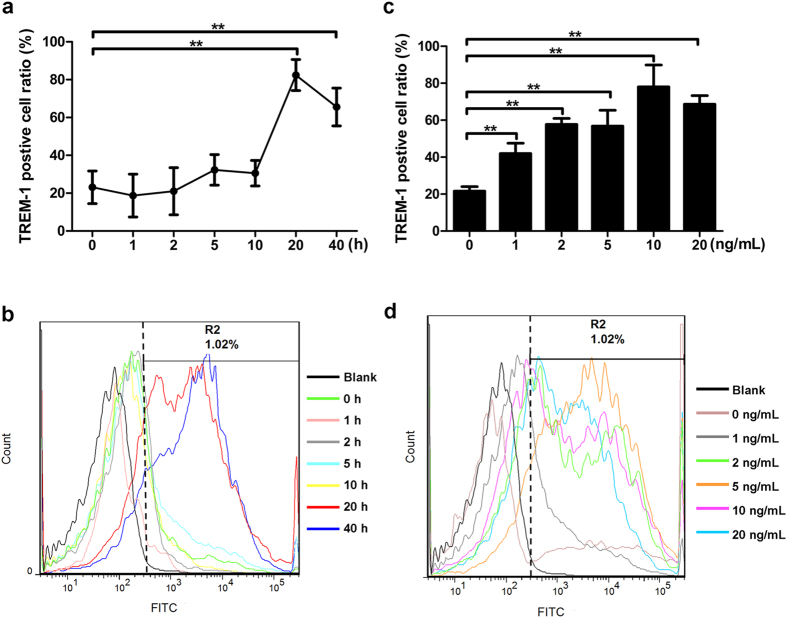
TGF-β1 increased TREM-1 expression in a time- and dose-dependent manner in mouse primary alveolar macrophages. (**a**,**b**) TREM-1 protein level on the surface of mouse primary alveolar macrophages after stimulation with 10 ng/mL TGF-β1 for various periods of time (0, 1, 2, 5, 10, 20, and 40 h) was tested by Flow cytometry. (**c**,**d**) The protein content of TREM-1 of macrophages following incubation with TGF-β1 at various concentrations (0, 1, 2, 5, 10, and 20 ng/mL) for 20 h was examined by Flow cytometry. Data were expressed as mean ± SD and were representative of 2 separate experiments performed in triplicate, **p < 0.01.

In order to further validate the effect of TGF-β1 on TREM-1 expression, the mouse primary alveolar macrophages were isolated and treated with 10 ng/mL TGF-β1 for various periods of time (0, 1, 2, 5, 10, 20, and 40 h) as well as TGF-β1 at various concentrations (0, 1, 2, 5, 10, and 20 ng/mL) for 20 h. The results showed that TREM-1 protein level was not altered following 1, 2, 5, and 10 h incubation with 10 ng/ml TGF-β1 (*p* > 0.05), but there was a distinct increase at 20 h and 40 h, with the highest expression level at 20 h after TGF-β1 stimulation in primary alveolar macrophages (*p* < 0.05) (Fig. 3a,b). Also, TREM-1 protein level elevated following treatment with all tested concentrations of TGF-β1 in a dose-dependent manner, with a peak expression at 10 ng/mL TGF-β1 in primary alveolar macrophages (*p* < 0.05) (Fig. 3c,d). These results indicated that TGF-β1 increases TREM-1 protein expression in a time- and dose-dependent manner in mouse primary alveolar macrophages, which were consistent with the effect of TGF-β1 on TREM-1 expression in mouse macrophages cell line.

### TGF-β1-Dependent Increase in TREM-1 Expression may be linked to AP-1 Expression in Mouse Macrophages

**Figure 4:**
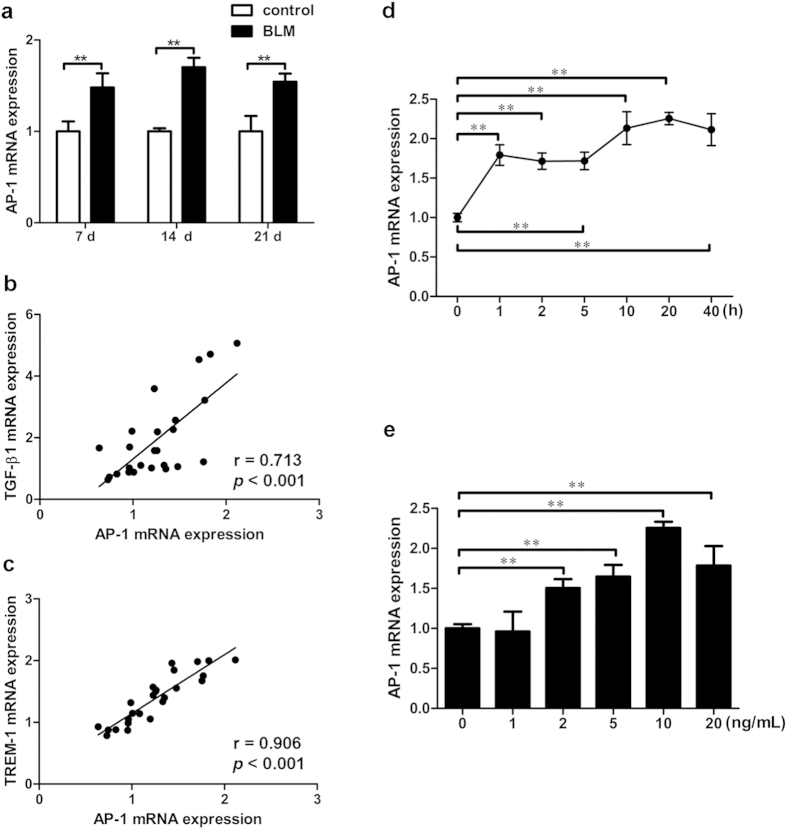
The TGF-β1 mediated increase in TREM-1 expression might be associated with AP-1 in mouse macrophages. Expression of AP-1 mRNA in lung tissues (**a**) was determined by real-time PCR. Correlations between AP-1 and TGF-β1 (**b**), between AP-1 and TREM-1 (**c**) by Pearson correlation analysis, the correlation coefficients were 0.773 and 0.773 (*p* < 0.01), respectively. The expression of AP-1 mRNA in 10 ng/ml TGF-β1 stimulated mouse macrophages at different time points (0, 1, 2, 5, 10, 20 and 40 h) and different concentrations for 20 h (0, 1, 2, 5, 10 and 20 ng/mL) (**d**,**e**) was detected by real-time PCR. Data were expressed as mean ± SD and were representative of 5 separate experiments performed in 8 mice or quadruplicate per group, **p < 0.01.

In order to assess the transcriptional level of AP-1 in the lungs of mice with pulmonary fibrosis, AP-1 mRNA in lung tissues was detected by real-time PCR. We found the expression of AP-1 mRNA levels was increased significantly on day 7, 14, and 21 in the BLM-treated group (*p* < 0.05) (Fig. 4a). In order to further analyze the relationship between AP-1 and TGF-β1 or TREM-1 in pulmonary fibrosis, Pearson Correlation analysis was performed, and the results showed that the AP-1 mRNA expression was positively related to TGF-β1 mRNA expression (r = 0.713, *p* < 0.05) (Fig. 4b) and TREM-1 mRNA expression (r = 0.906, *p* < 0.05) (Fig. 4c), respectively. In order to study any putative effect of TGF-β1 on AP-1 transcription levels, the expression of AP-1 mRNA was measured by real-time PCR after mouse macrophages were stimulated with 10 ng/ml TGF-β1 for various periods of time (0, 1, 2, 5, 10, 20 and 40 h), or various concentrations (0, 1, 2, 5, 10 and 20 ng/mL) for 20 h. Our results showed that expression of AP-1 mRNA was increased significantly 1 h after stimulation with 10 ng/ml TGF-β1 and increased expression of AP-1 mRNA was sustained up to 40 h post-stimulation (*p* < 0.05). Meanwhile, expression of AP-1 mRNA was increased significantly when 2, 5, 10, and 20 ng/mL of TGF-β1 were used to stimulate macrophages for 20 h, and the maximal induction of AP-1 mRNA expression was observed when 10 ng/mL TGF-β1 was used (Fig. 4d,e).

### Transcription Factor AP-1 was Involved in TGF-β1 Induced TREM-1 Expression in Mouse Macrophages

**Figure 5:**
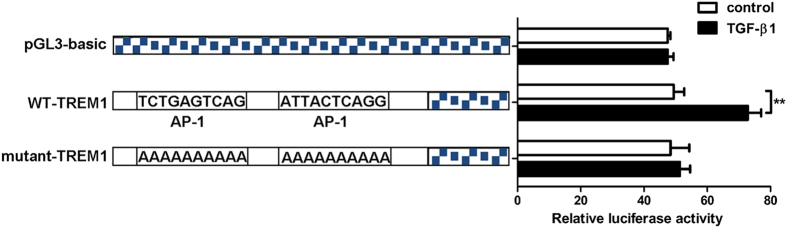
TGF-β1 stimulation increased the transcriptional activity of AP-1 at the TREM-1 promoter. Mouse macrophages were transfected with pGL3-basic vector (500 ng), or WT-TREM1 reporter plasmid (500 ng), or mutant-TREM1 reporter plasmid (500 ng), and the renilla luciferase expressing pRL-TK plasmid (1 ng, an internal control) and were incubated for 5 h. Cells were then incubated in the absence or presence of TGF-β1 (10 ng/mL) for 20 h. Firefly and renilla luciferase activities were detected using Varioskan Flash. The relative luciferase activities were standardized to renilla luciferase activity. Data were expressed as mean ± SD and were representative of 5 separate experiments performed in quadruplicate, **p < 0.01.

To further confirm the transcriptional control of AP-1 in the upregulation of TREM-1 by TGF-β1 in mouse macrophages, WT- and mutant-TREM-1 plasmids (containing mutations at the AP-1 promoter binding site) were constructed and the binding activity of AP-1 with TREM-1 promoter under TGF-β1 stimulation was measured by the dual-luciferase reporter assay system. The results showed that TGF-β1 did not stimulate luciferase expression in the pGL3-basic plasmid (empty plasmid) (*p* > 0.05), but significantly increased the relative luciferase activity in WT-TREM-1 plasmid (*p* < 0.05). No increase in relative luciferase activity was observed when the mutant-TREM–1 plasmid was used (*p* > 0.05) (Fig. 5).

## Discussion

The concept of the “genome storm” is a novel paradigm of human immune reactions after serious injury^1^. Research has found that over 80% of the transcriptome of white blood cell (WBC) changed within 4 to 12 h after severe injury, and that the changes were maintained for several weeks and even months after injuries^2^. The expression of genes involved in innate immunity as well as systemic immune and anti-inflammatory reactions was increased, while the expression of genes regulating adaptive immunity was decreased. These observations are in line with the concept of “nonresolving inflammation” and challenge the traditional “two-hit” model^1^. This new paradigm clearly suggests that therapies that limit the initial “genomic storm” within WBCs may be valuable in improving the prognosis of patients with severe injury^2^,^29^. Multiple proteins play their parts in amplifying the initial inflammatory response, including the upregulated TREM-1 during the “genomic storm” research, which may be attractive therapeutic targets. As an inflammatory amplificational factor^30^, TREM-1 comes into play in a variety of diseases, such as sepsis^31^, pneumonia^32^, lung cancer^33^, periodontitis^34^ and relapsing polychondritis^35^. However, there has been little study on TREM-1 in the pathogenesis of pulmonary fibrosis.

Pulmonary fibrosis is an irreversible and lethal interstitial lung disease with no effective pharmacological treatment^36^,^37^. BLM, an antibiotic, is commonly used for studying human pulmonary fibrosis in mouse and rat models. Following BLM treatment the lung undergoes significant biochemical, histological and physiological changes, which are similar to those of humans, leading to pulmonary fibrosis^12^,^36^. We found that TGF-β1 expression was positively correlated to the expression of TREM-1 significantly, and expression of the TREM-1 was upregulated in the lungs of mice with BLM-induced pulmonary fibrosis. These observations leaded us to further investigate the role of macrophages in this possible cross-talk feedback interaction between TGF-β1 and TREM-1 in BLM-induced pulmonary fibrosis. We observed the changes in TREM-1 expression following stimulation with TGF-β1 and found that the low levels of TREM-1 expression in the unstimulated state, while the expression of TREM-1 was increased significantly in a time- and dose-dependent manner upon stimulation with TGF-β1. The previous studies have shown that TGF-β1 is a central regulator in the processes of pulmonary fibrosis^38^, while TREM-1 can markedly increase the expression of TGF-β family genes. Therefore, the expression of TGF-β1 and TREM-1 is mutually synergistic and a positive feedback can be formed that speeds up the development and progression of pulmonary fibrosis. It is worth noting that TGF-β1 is historically considered as a typical anti-inflammatory factor^39^,^40^, but our experiments showed that TGF-β1 exhibits pro-inflammatory effects by dramatically increasing the expression of the inflammatory amplifier TREM-1. This effect may be attributed to the different effects of TGF-β1 in distinct cell types and disease models.

AP-1 is a transcription factor, a heteromultimeric protein that is composed of proteins from the Jun (c-Jun, Jun-B, Jun-D) and Fos (c-Fos, Fos-B, Fra-1, Fra-2) families of proteins. AP-1 works by controlling a broad range of responses to different stimuli and regulating the expression of genes involved in oxidative stress, inflammatory reactions, cell growth, and cell remodeling^41^,^42^. One previous study has shown that AP-1 can increase TREM-1 transcription induced by LPS in mouse macrophages^28^, indicating that AP-1 may be a transcription regulator in LPS-induced expression of TREM-1. AP-1 transcriptional activity is regulated by TGF-β1 *in vivo* and *in vitro*^43^. These findings suggest that AP-1 may be related to the upregulation of TREM-1 upon stimulation by TGF-β1. Therefore, we determined the expression of AP-1 in BLM-induced pulmonary fibrosis and we found that the expression of AP-1 mRNA was increased significantly in BLM-treated mice as well as macrophages suggesting an important role of AP-1 in BLM-induced pulmonary fibrosis. We also found that the binding activity of AP-1 with TREM-1 promoter is increased significantly upon TGF-β1 stimulation, but this pattern was not observed when the AP-1 binding sites in the TREM-1 promoter were mutated to alanines. To our knowledge, this is the first observation that reveals AP-1-mediated transcriptional control in TGF-β1-induced upregulation of the TREM-1 in cultured macrophages.

It should be noted that no functional data are performed in this mechanistic study. It is not clear whether modification of the expression of TREM-1 and TGF-β1 affects pulmonary function or progression of the pulmonary fibrosis. Further studies are warranted to illustrate the functional consequences of this interplay between TREM-1 and TGF-β1. Interestingly, a new TREM-1 inhibitor LR12 has just been discovered^29^, it is worthwhile to investigate whether LR12 can ameliorate BLM-induced pulmonary fibrosis and therefore improve functional outcome in this animal model.

In conclusion, we found that 1) the expression of TREM-1 was increased significantly in a mouse model of BLM-induced pulmonary fibrosis; 2) TGF-β1 increased expression of the TREM-1 by interfering AP-1 transcriptional pathway in mouse macrophages. TGF-β1 may form a positive feedback with TREM-1 and accelerate the progression of pulmonary fibrosis. These findings may provide new mechanistic insights into the pathogenesis of pulmonary fibrosis.

## Additional Information

**How to cite this article**: Peng, L. *et al.* TGF-β1 Upregulates the Expression of Triggering Receptor Expressed on Myeloid Cells 1 in Murine Lungs. *Sci. Rep.* **6**, 18946; doi: 10.1038/srep18946 (2016).

## Supplementary Material

Supplementary Information

## Figures and Tables

**Table 1:** Oligonucleotide primers used for the PCR-based site-directed mutagenesis.

sites of mutagenesis		primer sequences
AP-1 (1)	sense	GTAGTGAAAAAAAAAAGGTTTCTATTCCTGCACAAAC
	anti-sense	GAAACCTTTTTTTTTTCACTACCTCAGACACATTCC
AP-1 (2)	sense	CTGTCTCAAAAAAAAAAAAGCTAAATGGGCTGGGGTAG
	anti-sense	CATTTAGCTTTTTTTTTTTTGAGACAGAGGCCTGTTGG
