# Supplementary material for: TGF-β1 Upregulates the Expression of Triggering Receptor Expressed on Myeloid Cells 1 in Murine Lungs
Source: Sci Rep. 2016 Jan 7;6:18946. doi: 10.1038/srep18946 (PMC4704059; doi:10.1038/srep18946)
Supplement: Supplementary Information [file srep18946-s1.doc]

**TGF-β1 Upregulates the Expression of Triggering Receptor Expressed on Myeloid Cells 1 in Murine Lungs**

Li Peng^1^, Yong Zhou^1^, Liang Dong^1^, Rui-Qi Chen^1^, Guo-Ying Sun^1^, Tian Liu^1^, Wen-Zhuo Ran^1^, Xiang Fang^2^, Jian-Xin Jiang^3^*, Cha-Xiang Guan^1^*

^1^ Department of Physiology, Xiangya School of Medicine, Central South University, Changsha, China; ^2^ Department of Neurology, University of Texas Medical Branch, Galveston, TX 77555, USA; ^3^ State Key Laboratory of Trauma, Burns, and Combined Injury, Research Institute of Surgery, Daping Hospital, Third Military Medical University, Chongqing, China

**Running title: TGF-β1 and TREM-1 expression**

*** Corresponding author:**

Prof. Cha-Xiang Guan

Department of Physiology, Xiangya School of Medicine, Central South University

Changsha, Hunan 410078, China

Tel: +86-731-82355051; Fax: +86-731-82355056; E-mail: guanchaxiang@csu.edu.cn

Prof. Jian-Xin Jiang

State Key Laboratory of Trauma, Burns, and Combined Injury

Research Institute of Surgery, Daping Hospital

Third Military Medical University

Chongqing, Sichuan 400042, China

Tel: +86-23-68757401; Fax: +86-23-68706323; E-mail: hellojjx@126.com


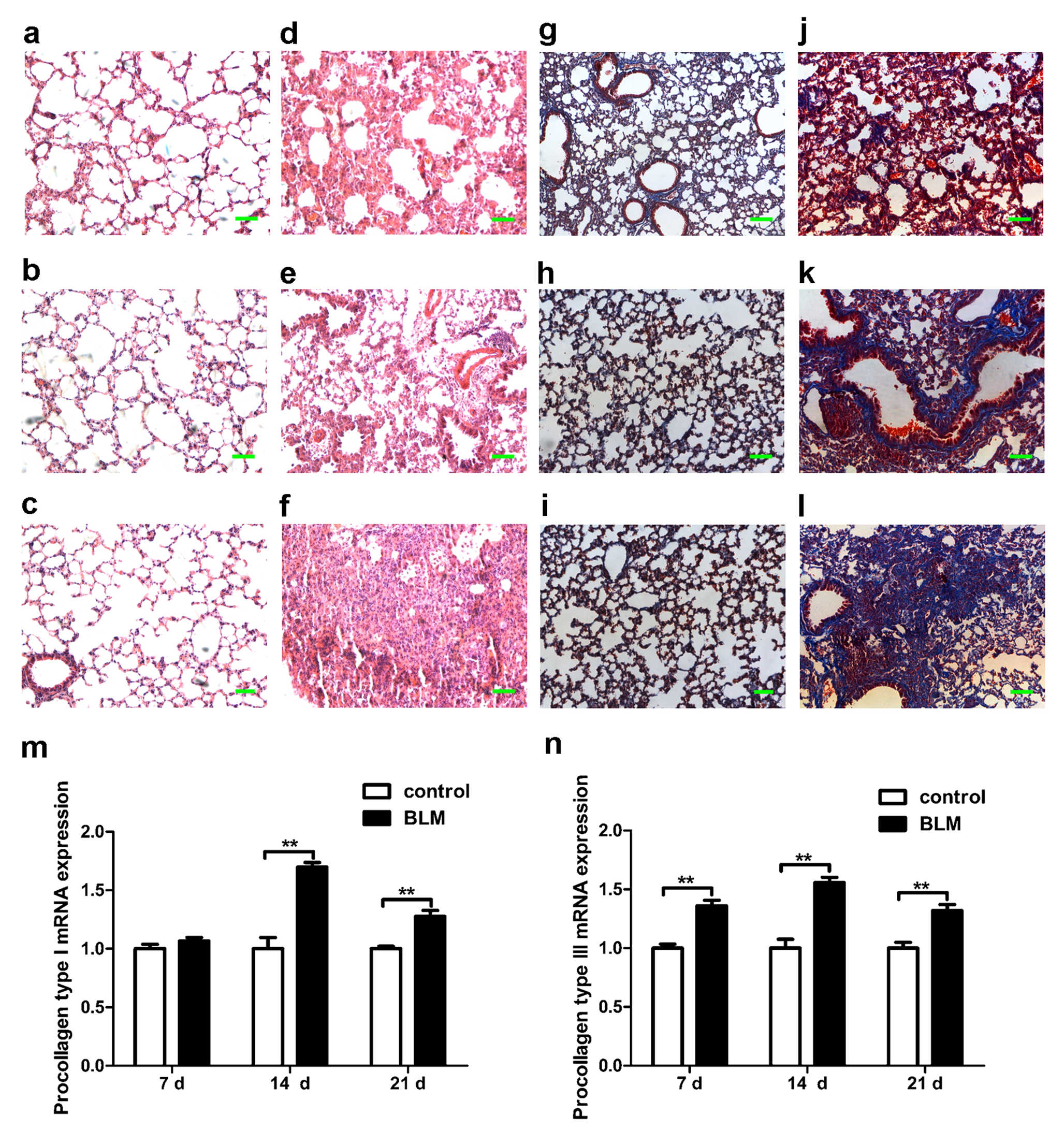


**Figure S1** | **Establishment of a** **mouse pulmonary fibrosis model.** Typical images of HE-stained (**a-f**) and Masson-stained (**g-i**) lung tissues of control and BLM mice. **a, g**: control group 7 d; **b, h**: control group 14 d; **c, i**: control group 21 d; **d, j**: BLM group 7 d; **e, k**: BLM group 14 d; **f, i**: BLM group 21 d. Scale bar = 100 um. Collagen fibers appear blue in the lung tissues after Masson staining. The mRNA levels of type I (**m**) and III (**n**) procollagen in the lung tissues were determined by real-time PCR. Data were expressed as the mean ± SD with 8 mice per group, *******p*<0.01*.*


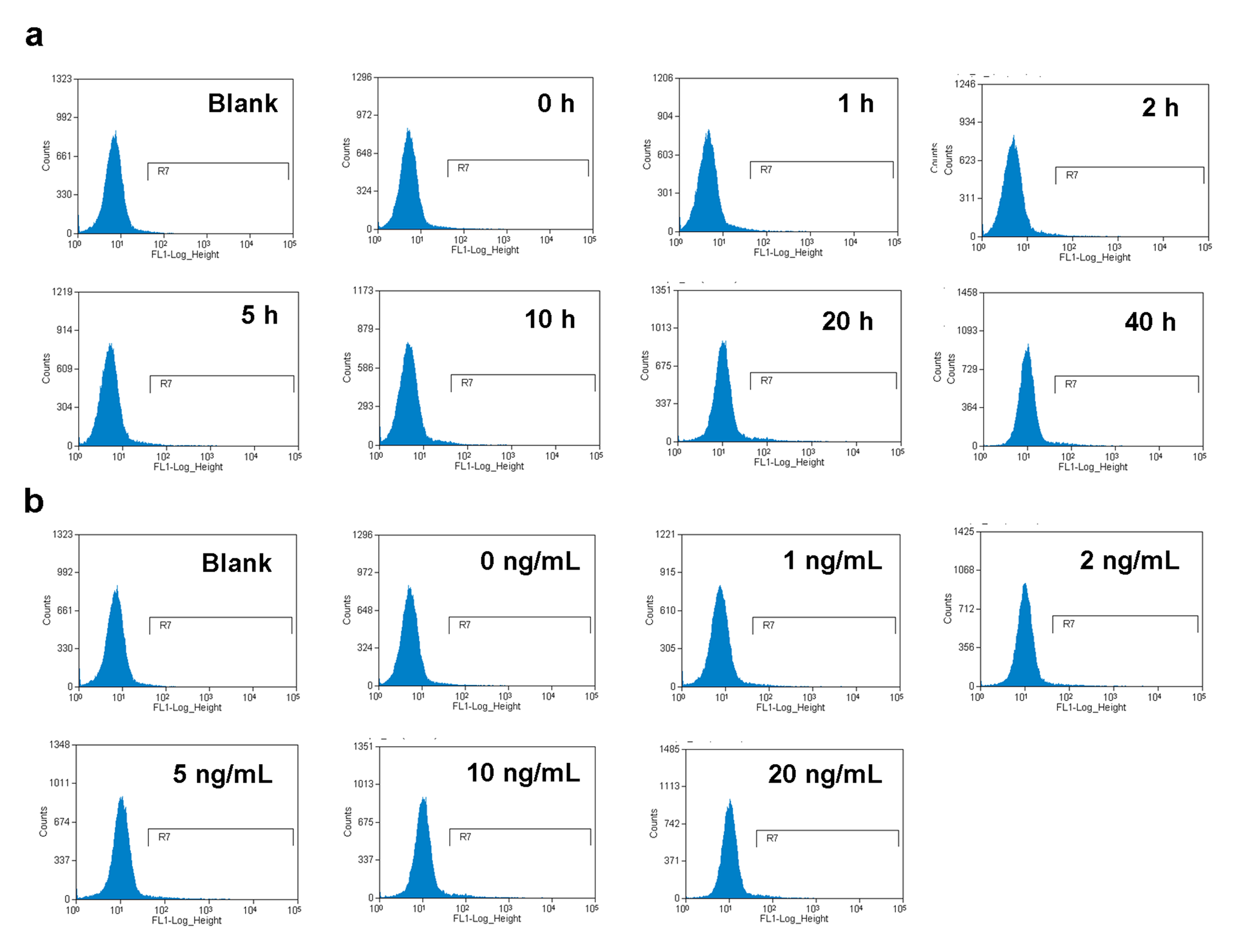


**Figure S2** | **TGF-β1 increased TREM-1 expression in a time- and dose-dependent manner in mouse macrophages line.** (**a**) Representative Flow cytometry graphs were from TREM-1 protein level on the surface of mouse macrophages line after stimulation with 10 ng/mL TGF-β1 for various periods of time (0, 1, 2, 5, 10, 20, and 40 h). (**b**) Typical Flow cytometry graphs represented the protein content of TREM-1 on the surface of mouse macrophages line following incubation with TGF-β1 at various concentrations (0, 1, 2, 5, 10, and 20 ng/mL) for 20 h was examined by Flow cytometry.


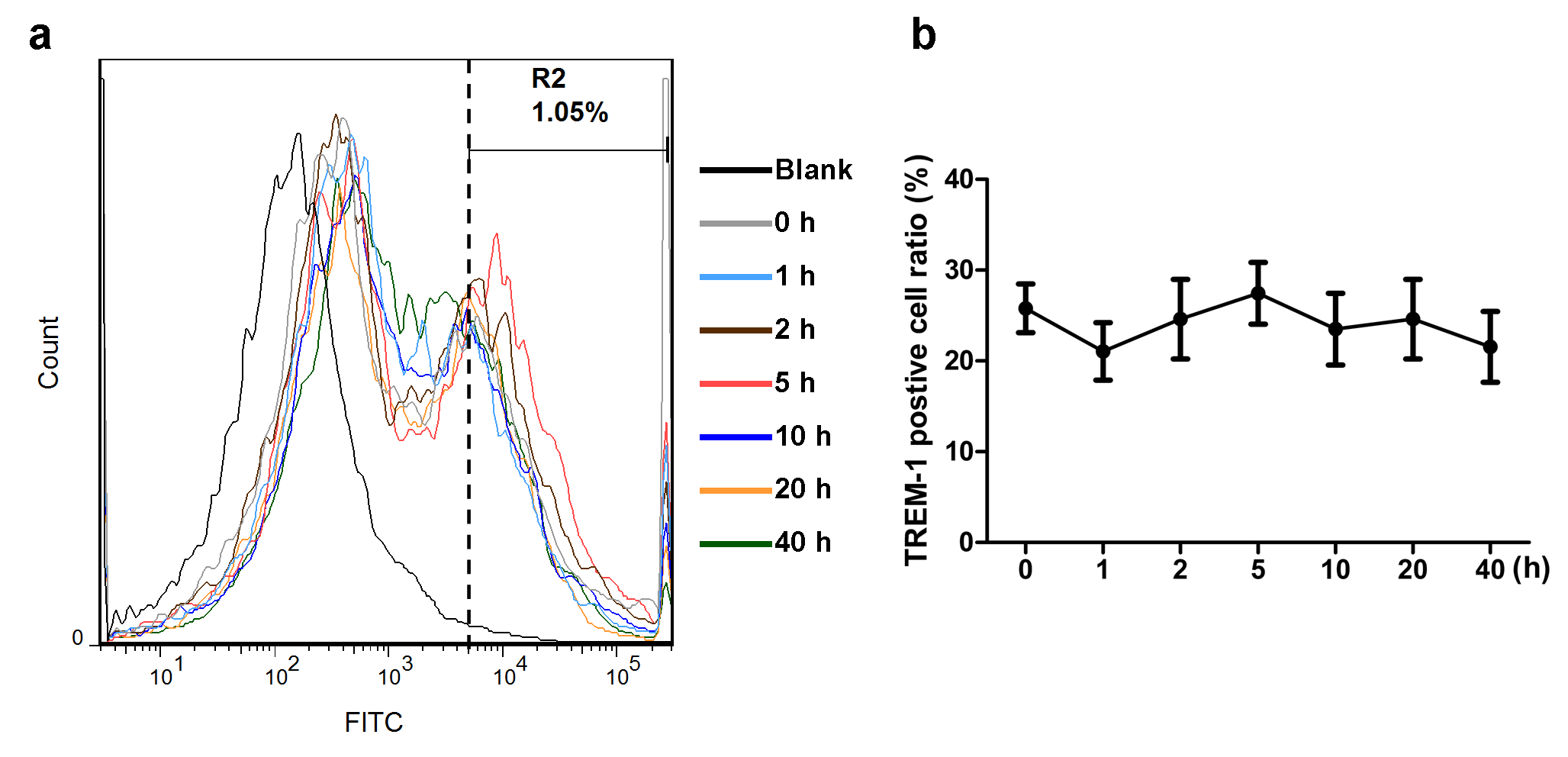


**Figure S3** | **TREM-1 expression does not increase with time in mouse macrophages with no-added TGF-β.** TREM-1 protein level does not increase with time (0, 1, 2, 5, 10, 20, and 40 h) in primary mouse alveolar macrophages.
